# Animal models of chronic tympanic membrane perforation: in response to plasminogen initiates and potentiates the healing of acute and chronic tympanic membrane perforations in mice

**DOI:** 10.1186/2001-1326-3-5

**Published:** 2014-03-26

**Authors:** Allen Y Wang, Yi Shen, Jeffrey T Wang, Robert H Eikelboom, Rodney J Dilley

**Affiliations:** 1 Ear Sciences Centre, School of Surgery, The University of Western Australia, 35 Stirling Highway, Nedlands, WA 6009, Australia; 2 Ear Science Institute Australia, Subiaco, WA, Australia; 3 Department of Otolaryngology, Head, Neck and Skull Base Surgery, Sir Charles Gairdner Hospital, Nedlands, WA, Australia; 4 Department of Otolaryngology, Head and Neck, Ningbo Lihuili Hospital (Ningbo Medical Centre); School of Medicine, Ningbo University, Ningbo, Zhejiang, China; 5 Department of Speech-Language Pathology and Audiology, University of Pretoria, Pretoria, South Africa

**Keywords:** Tympanic membrane perforation, Chronic, Animal model, Otology, Plasminogen, Gene deficiency

## Abstract

Tympanic membrane perforations (TMP) are relatively common but are typically not treated in their acute stage, as most will heal spontaneously in 7–10 days. Those cases which fail to heal within 3 months are called chronic TMP which attract surgical intervention (e.g. myringoplasty), typically with a temporalis fascia autograft. New materials for the repair of chronic TMP are being developed to address deficiencies in the performance of autografts by undergoing evaluation in animal models prior to clinical study. However, there is currently a lack of ideal chronic TMP animal models available, hindering the development of new treatments. Various techniques and animal species have been investigated for the creation of chronic TMP with varied success. In the present commentary, we bring to the attention of readers the recent report by Shen et al. in Journal of Translational Medicine. The study reported the creation of a chronic TMP animal model in plasminogen gene deficient mice. However, the short observation time (9, 19 days), lack of success rate and the scarcity of solid evidence (e.g. otoscopic & histologic images) to confirm the chronicity of TMP warrant a more thorough discussion.

## Background

Tympanic membrane perforations (TMP) are a common problem in otology, resulting from trauma, infection (e.g., otitis media) or a sequel after extrusion of tympanostomy tubes. A major proportion of the acute TMP heals spontaneously in 7–10 days [1,2] without further intervention by epithelial migration, fibroblastic activity and vascular proliferation. A minority of cases fails to heal within 3 months which are called chronic TMP associating with morbidities such as conductive hearing loss, recurrent chronic infections and cholesteatoma formation. Chronic TMP attract surgical intervention (e.g. myringoplasty), typically with a temporalis fascia autograft.

New materials for the repair of chronic TMP are being developed to address deficiencies in the performance of autografts by undergoing evaluation in animal models prior to clinical study [3]. These studies have been investigated on a variety of animal models, including rat [4,5], mice [6], chinchilla [7], guinea pig [8,9] and dog [10]. However their relevance has been hampered by the utilization of acute TMP models rather than chronic. The major inadequacy of the acute model is that up to 90% [1,2] heal spontaneously without intervention and therefore accelerating the healing of acute TMP is of little practical value. Hence, a chronic TMP animal model would have more clinical relevance [11].

The lack of an ideal chronic TMP animal model was initially brought to attention in 2007 [12], as this issue hinders the development of new treatment. Since then, there have been additional studies in the literature involving creation of chronic TMP animal model associated with various techniques, animal species and success rates. However, whether these existing methods do in fact create a ‘true’ chronic TMP is still controversial.

## Commentary

We read with great interest the recent research article by Shen et al. [13] investigating the efficacy of injections of plasminogen (plg) on the healing of both acute and chronic tympanic membrane perforations (TMP) in mice with plg gene deficiency (plg^–/–^). This intervention significantly improved healing rates of chronic TMP in plg^–/–^ mice and indicates an important role of plasminogen in TMP healing.

We would like to draw attention to the labeling of this model as a chronic perforation by Shen et al. [13], and the indicated potential for translating plasminogen injection treatment to clinical use. The tympanic membranes were perforated on day 0, left untreated for a period of 9 days, and thereafter treatments applied for comparison of effects on healing rate over a further period of 10 days. However, the most popular consensus in the current literature defines the minimum duration of chronic TMP patency to be at least 8 weeks in animal models [14-24]. Can this short time period of 9 days be defined as chronic or rather as delayed? Indeed, many publications of chronic TMP animal model have conservatively defined their TMP failing to reach the maximum patency time up to 8 weeks as delayed healing [4,25-31], including the recently reported study on mice with deficiency in urokinase-type plg activators [25].

More detailed information may help to resolve this question, specifically to the methodology of assessing a chronic TMP animal model. The success rate of this chronic TMP method (i.e. number of successful chronic TMP before plg injection divided by the total number of ears initially attempted) was not provided. Thus, it is difficult to assess the reliability and stability of this particular chronic method. This study has employed an animal model (i.e. plg^–/–^ mice) previously described by Li et al. [32] demonstrating that in plg^–/–^ mice, 2 of 10 TMP (success rate of 80%) have closed on day 8; 21 of 26 TMP (success rate of 19%) have healed by day 50 (i.e. just after 7 weeks) and all TMP have closed by day 143. Given the relatively low success rate at day 50, this seems to be a delayed model under the conservative definition, rather than a chronic model.

It may be of benefit to illustrate chronicity of the TMP with morphologic evidence, such as otoscopic and histologic images. Otoscopic examination is necessary to evaluate the patency of a chronic TMP with typical features of thickened and opaque margins compared to an acute TMP. Microscopically, a chronic TMP is featured by squamous epithelium growing over the perforation edge to join with the medial mucosal layer [7,33]. This evidence was not included in this article to confirm that chronicity of TMP was achieved before the intervention of plg injections. Previous studies with success have presented this important piece of histologic evidence [7,10,14,16]. Thus, whether ‘true’ chronic TMP were produced in this mouse model given the absence of otoscopic and histologic proof is uncertain.

## Discussion

The absence of an ideal chronic TMP animal model in the current literature has been brought to attention in recent years [3,11,12], as this issue hinders development of treatments for chronic TMP in a clinical setting. Since then, there have been several studies in the literature involving creation of chronic TMP animal models associated with various techniques (e.g. infolding technique [7,14,16], thermal injury + reperforation [22,23,34], mitomycin C + steroids [21,24,35]), animals (e.g. chinchilla, rat, guinea pig, mouse) and success rates. However, whether these existing methods do in fact create a ‘true’ chronic TMP is still controversial [36,37]. In addition, in terms of TMP healing, there is a significant discrepancy between an acute and chronic TMP in terms of their underlying healing mechanisms and responses to the same treatment [9,38]. A major proportion of acute TMP in both human and rodents heal spontaneously without any intervention within 7–10 [1,2] and 5–7 days [39,40] respectively via otoscopic observation. Thus, mislabeling an actual acute TMP model as chronic may give an ambiguous interpretation.

## Conclusion

In conclusion, we value the potential of plg^–/–^ mice as an animal model of TMP healing mechanisms [13], but an observation period of at least 8 weeks before treatment is commenced may ensure a chronic condition is being treated. The outcome of this novel injection of plg for TMP repair seems ultimately to be promising for clinical therapy. However, caution needs to be paid to the reliability of this animal model to provide an accurate prediction of efficacy for chronic TMP, otherwise the success of future transition to human clinical trials may be doubtful and even potentially risky.

## Abbreviations

Plg: Plasminogen; plg^–/–^: Plasminogen deficient; TMP: Tympanic membrane perforation.

## Competing interests

The authors declare that they have no competing interests.

## Authors’ contribution

AYW, YS, JTW, RHE and RJD made intellectual contributions and drafted the manuscript. All authors read and approved the final manuscript.

## References

[B1] Lindeman P Edström S Granström G Jacobsson S von Sydow C Westin T Aberg B Acute traumatic tympanic membrane perforations. Cover or observe? Arch Otolaryngol Head Neck Surg 1987 3 1285 1287 3675893 10.1001/archotol.1987.01860120031002

[B2] Berger G Nature of spontaneous tympanic membrane perforation in acute otitis media in children J Laryngol Otol 1989 3 1150 1153 2614234 10.1017/s0022215100111247

[B3] Teh BM Marano RJ Shen Y Friedland PL Dilley RJ Atlas MD Tissue engineering of the tympanic membrane Tissue Eng Part B Rev 2013 3 116 132 23031158 10.1089/ten.TEB.2012.0389

[B4] O’Reilly RC Goldman SA Widner SA Cass SP Creating a s tympanic membrane perforation using mitomycin C Otolaryngol Head Neck Surg 2001 3 40 45 11228450 10.1067/mhn.2001.112199

[B5] Shen Y Redmond SL Teh BM Yan S Wang Y Zhou L Budgeon CA Eikelboom RH Atlas MD Dilley RJ Zheng M Marano RJ Scaffolds for tympanic membrane regeneration in rats Tissue Eng Part A 2013 3 657 668 23092139 10.1089/ten.tea.2012.0053 PMC3566677

[B6] Prestwich AH Li J Eriksson PO Ny T Berggren D Hellström S Lack of plasminogen does not alter the early inflammatory response following a tympanic membrane perforation: a study in plasminogen-deficient mice Acta Otolaryngol 2008 3 1294 1302 18781446 10.1080/00016480701361996

[B7] Amoils CP Jackler RK Milczuk H Kelly KE Cao K An animal model of chronic tympanic membrane perforation Otolaryngol Head Neck Surg 1992 3 47 55 1734366 10.1177/019459989210600127

[B8] Jassir D Odabasi O Gomez-Marin O Buchman CA Dose-response relationship of topically applied mitomycin C for the prevention of laser myringotomy closure Otolaryngol Head Neck Surg 2003 3 471 474 14595268 10.1016/S0194-59980301394-9

[B9] Shen Y Redmond SL Teh BM Yan S Wang Y Atlas MD Dilley RJ Zheng M Marano RJ Tympanic membrane repair using silk fibroin and acellular collagen scaffolds Laryngoscope 1976–1982 3 123 10.1002/lary.23940 23536496

[B10] Truy E Disant F Morgon A Chronic tympanic membrane perforation: an animal model Am J Otol 1995 3 222 225 8572123

[B11] Hong P Bance M Gratzer PF Repair of tympanic membrane perforation using novel adjuvant therapies: a contemporary review of experimental and tissue engineering studies Int J Pediatr Otorhinolaryngol 2013 3 3 12 23044356 10.1016/j.ijporl.2012.09.022

[B12] Santa Maria PL Atlas MD Ghassemifar R Chronic tympanic membrane perforation: a better animal model is needed Wound Repair Regen 2007 3 450 458 17650087 10.1111/j.1524-475X.2007.00251.x

[B13] Shen Y Guo Y Wilczynska M Li J Hellström S Ny T Plasminogen initiates and potentiates the healing of acute and chronic tympanic membrane perforations in mice J Transl Med 2014 3 5 24393366 10.1186/1479-5876-12-5 PMC3895791

[B14] Parekh A Mantle B Banks J Swarts JD Badylak SF Dohar JE Hebda PA Repair of the tympanic membrane with urinary bladder matrix Laryngoscope 2009 3 1206 1213 19358244 10.1002/lary.20233 PMC3003594

[B15] Spiegel JH Kessler JL Tympanic membrane perforation repair with acellular porcine submucosa Otol Neurotol 2005 3 563 566 16015147 10.1097/01.mao.0000169636.63440.4e

[B16] Deng Z Wu J Qiu J Wang J Tian Y Li Y Jin Y Comparison of porcine acellular dermis and dura mater as natural scaffolds for bioengineering tympanic membranes Tissue Eng Part A 2009 3 3729 3739 19519275 10.1089/ten.TEA.2008.0460

[B17] Lee AJ Jackler RK Kato BM Scott NM Repair of chronic tympanic membrane perforations using epidermal growth factor: progress toward clinical application Am J Otol 1994 3 10 18 8109618

[B18] Laidlaw DW Costantino PD Govindaraj S Hiltzik DH Catalano PJ Tympanic membrane repair with a dermal allograft Laryngoscope 2001 3 702 707 11359143 10.1097/00005537-200104000-00025

[B19] Amoils CP Jackler RK Lustig LR Repair of chronic tympanic membrane perforations using epidermal growth factor Otolaryngol Head Neck Surg 1992 3 669 683 1437205 10.1177/019459989210700509

[B20] Dvorak DW Abbas G Ali T Stevenson S Welling DB Repair of chronic tympanic membrane perforations with long-term epidermal growth factor Laryngoscope 1995 3 1300 1304 8523981 10.1288/00005537-199512000-00007

[B21] Choi SJ Kim WS Kim JH Lee JB Choo OS Chung JH Choung YH Efficient treatment of chronic tympanic membrane perforations in animal models by chitosan patch scaffolds Tissue Eng Regen Med 2011 3 141

[B22] Wieland AM Sundback CA Hart A Kulig K Masiakos PT Hartnick CJ Poly(glycerol sebacate)-engineered plugs to repair chronic tympanic membrane perforations in a chinchilla model Otolaryngol Head Neck Surg 2010 3 127 133 20620631 10.1016/j.otohns.2010.01.025

[B23] Sundback CA McFadden J Hart A Kulig KM Wieland AM Pereira MJ Pomerantseva I Hartnick CJ Masiakos PT Behavior of poly(glycerol sebacate) plugs in chronic tympanic membrane perforations J Biomed Mater Res B Appl Biomater 1943–1954 3 100 10.1002/jbm.b.32761 22821822

[B24] Cui XD Lu F Zhao X Creating an animal model of rats for chronic persistent tympanic membrane perforation J Shanghai Medica (University) 2005 3 033

[B25] Shen Y Guo Y Du C Wilczynska M Hellström S Ny T Mice deficient in urokinase-type plasminogen activator have delayed healing of tympanic membrane perforations PLoS One 2012 3 e51303 23236466 10.1371/journal.pone.0051303 PMC3517469

[B26] Kaftan H Reuther L Miehe B Hosemann W Herzog M Delay of tympanic membrane wound healing in rats with topical application of a tyrosine kinase inhibitor Wound Repair Regen 2008 3 364 369 18471254 10.1111/j.1524-475X.2008.00375.x

[B27] Kaftan H Reuther L Miehe B Hosemann W Beule A Inhibition of fibroblast growth factor receptor 1: influence on tympanic membrane wound healing in rats Eur Arch Otorhinolaryngol 2012 3 87 92 21590482 10.1007/s00405-011-1627-6

[B28] Kaftan H Vogelgesang S Lempas K Hosemann W Herzog M Inhibition of epidermal growth factor receptor by erlotinib: wound healing of experimental tympanic membrane perforations Otol Neurotol 2007 3 245 249 17255893 10.1097/01.mao.0000244366.24449.db

[B29] Kaftan H Reuther L Miehe B Hosemann W Herzog M The influence of inhibition of the epidermal growth factor receptor on tympanic membrane wound healing in rats Growth Factors 2010 3 286 292 20166887 10.3109/08977191003620238

[B30] Cakir BO Dadaş B Başak T Cinar U Ozdoğan HC Uslu B Turgut S Effect of topical 5-fluorouracil on closure time of myringotomies created by a radiofrequency surgical unit in guinea pigs Otol Neurotol 2002 3 146 151 11875341 10.1097/00129492-200203000-00007

[B31] Ramalho JR Bento RF Healing of subacute tympanic membrane perforations in chinchillas treated with epidermal growth factor and pentoxifylline Otol Neurotol 2006 3 720 727 16868521 10.1097/01.mao.0000226316.04940.f9

[B32] Li J Eriksson PO Hansson A Hellström S Ny T Plasmin/plasminogen is essential for the healing of tympanic membrane perforations Thromb Haemost 2006 3 512 519 17003931

[B33] Dunlap AM Schuknecht HF Closure of perforations of the tympanic membrane Laryngoscope 1947 3 479 490 20256383

[B34] Soumekh B Hom DB Levine S Juhn SK Antonelli PJ Treatment of chronic tympanic-membrane perforations with a platelet-derived releasate Am J Otol 1996 3 506 511 8841694

[B35] Seonwoo H Kim SW Kim J Chunjie T Lim KT Kim YJ Pandey S Choung PH Choung YH Chung JH Regeneration of chronic tympanic membrane perforation using an EGF-releasing chitosan patch Tissue Eng Part A 2013 3 2097 2107 23627815 10.1089/ten.tea.2012.0617 PMC3725942

[B36] Santa Maria PL In response to: Regeneration of chronic tympanic membrane perforation using an EGF-releasing chitosan patch Tissue Eng Part A 2013 3 2109 2110 23859315 10.1089/ten.TEA.2013.0351

[B37] Seonwoo H Kim SW Kim J Chunjie T Lim KT Kim YJ Choung PH Choung YH Chung JH Response to “letter to the editor” written by Peter Luke Santa Maria, MBBS, PhD Tissue Eng Part A 2013 3 2110 2111 23865585 10.1089/ten.TEA.2013.0410

[B38] Gladstone HB Jackler RK Varav K Tympanic membrane wound healing An overview. Otolaryngol Clin North Am 1995 3 913 932 8559580

[B39] Santa Maria PL Redmond SL Atlas MD Ghassemifar R Histology of the healing tympanic membrane following perforation in rats Laryngoscope 2061–2070 3 120 10.1002/lary.20998 20824636

[B40] Clawson JP Litton WB The healing process of tympanic membrane perforations Trans Am Acad Ophthalmol Otolaryngol 1971 3 1302 1312 5151810

